# Environmentally persistent pathogens present unique challenges for studies of host–pathogen interactions: Reply to Field (2018)

**DOI:** 10.1002/ece3.4035

**Published:** 2018-05-08

**Authors:** Christina M. Davy, Michael E. Donaldson, Craig K. R. Willis, Barry J. Saville, Liam P. McGuire, Heather Mayberry, Alana Wilcox, Gudrun Wibbelt, Vikram Misra, Christopher J. Kyle

**Affiliations:** ^1^ Environmental and Life Sciences Graduate Program Trent University Peterborough ON Canada; ^2^ Wildlife Research and Monitoring Section Ontario Ministry of Natural Resources and Forestry Peterborough ON Canada; ^3^ Forensic Science Department Trent University Peterborough ON Canada; ^4^ Department of Biological Sciences Texas Tech University Lubbock TX USA; ^5^ Leibniz Institute of Zoo and Wildlife Research Berlin Germany; ^6^ Department of Microbiology Western College of Vet. Medicine University of Saskatchewan Saskatoon SK Canada

## Abstract

**Linked article**: https://doi.org/10.1002/ece3.4034

We thank Field (2018) for his comments on Davy et al. (2017), “The Other White‐Nose Syndrome Transcriptome: tolerant and susceptible hosts respond differently to the pathogen *Pseudogymnoascus destructans”,* where we reported the outcomes of experimental exposure of little brown bats, *Myotis lucifugus,* and greater mouse‐eared bats, *Myotis myotis,* to *P. destructans*. Field (2018) raises three key points: (1) that the putatively tolerant species in our experiment (*M. myotis*) did not develop infection, (2) that the reported qPCR results do not confirm the presence of the pathogen at the time of sampling, and (3) that the *M. myotis* tissue used for RNA sequencing did not contain the pathogen, *P. destructans*. These points highlight the unique challenges of studying host–pathogen interactions in environmentally persistent pathogens, and we welcome the opportunity to explicitly discuss these challenges.

## 1 *MYOTIS MYOTIS* EXPERIMENTALLY EXPOSED TO *P. DESTRUCTANS* DID NOT DEVELOP CLINICAL INFECTIONS

The bats in our experiment were exposed to a controlled dose of a virulent, viable pathogen, under conditions in which the same pathogen demonstrated growth and virulence on another host species, but did not develop infections. Therefore, our results provide an opportunity to quantify response (or lack thereof, in our case) to pathogen exposure, in the absence of disease, in a species (*M. myotis*) that is sometimes (but not always) resistant or tolerant to *P. destructans*. This point is clearly made in our study, in which we defined the treatment as “exposed to *P. destructans”*. We agree that our single use of the word “infection” in the Discussion should have read “exposed”, which is how we described the treatment of *M. myotis* in all other instances. In our discussion, we proposed experimental designs that could account for individual variation in response to *P. destructans* or WNS and that could be used to explore the temporal shift in responses of exposed individuals that develop or do not develop clinical disease. We are currently completing one such experiment, and we look forward to similar upcoming publications from Field et al.

## 2 QUANTITATIVE PCR ONLY DETECTED LOW PATHOGEN LOADS ON THREE EXPOSED *M. MYOTIS*

Field (2018) expressed skepticism that our positive qPCR results from three exposed *M. myotis* actually indicated pathogen presence, because one of these results was at the edge of the detection limit for the qPCR assay (*Ct* = 40). We appreciate the chance to clarify this point. We submitted swabs from each bat to the Pathogen & Microbiome Institute at Northern Arizona University, where they were tested with the qPCR described by Muller et al. (2013). This assay included negative controls, which were all confirmed negative, and a series of three dilutions of positive control (a quantified standard of isolate *P. destructans* 20631‐21; https://www.atcc.org/Products/All/MYA-4855D.aspx; K. Parise, personal communication). Each plate was run in duplicate in order to confirm positives and pick up any low‐level positives. We reported the absolute range of *Ct* values for the three *M. myotis* that tested positive, but the paired *Ct* values from the three Pd‐positive bats were 38.289/40.068; 36.998/38.598; and 33.064/33.183. Thus, each of these bats met the currently accepted threshold for qPCR detection of *P. destructans* (Langwig et al., 2015; Moore et al., 2017), but Field's point raises a pervasive challenge in the study of WNS.

Quantification of pathogen loads through qPCR is relatively simple in some systems, especially those where the pathogen is internal and relatively evenly distributed through the target tissue. Examples include qPCR quantification of *Plasmodium* sporozoite abundance in mosquito midguts and salivary glands (Emami, Ranford‐Cartwright, & Ferguson, 2017) and quantification of pathogen loads of the bacterium *Weissella ceti* in rainbow trout (*Oncorhynchus mykiss*; Snyder, Hinshaw, & Welch, 2015).

The interpretation of qPCR swab data in relation to WNS is a challenge for all research groups, because exposure (the presence of fungus on the wings) is not synonymous with infection. Furthermore, the pathogen is not distributed evenly across the wing, so swabs from different parts of the wing may pick up different concentrations of the pathogen. Ironically, swabbing the bats in our study for qPCR before sampling them for RNA sequencing may have reduced the detection probability of *P. destructans* during RNA sequencing. This challenge is not specific to our study. Examples from other excellent studies include putatively unexposed bats that produce borderline “positive” qPCR results, clinically infected bats considered as a single “treatment” group with qPCR‐based pathogen loads that range across orders of magnitude, and clinically infected bats that test negative for the pathogen based on qPCR of wing swabs (Field et al., 2015; Moore et al., 2017). Similar challenges occur in studies of snake fungal disease (Allender et al., 2015), where clinical infection may not correlate with pathogen detection based on skin swabs.

## 3 *PSEUDOGYMNOASCUS DESTRUCTANS* DID NOT INVADE THE TISSUE OF EXPOSED *M. MYOTIS*

Quantification of pathogen loads is essential for robust comparisons among infected individuals, and we have quantified pathogen load and gene expression in vitro and in infected bats, where our data permit (Davy et al., 2017; SI; Donaldson et al., 2018). However, our *M. myotis* did not develop infections, despite the confirmed superficial persistence of the pathogen on the wings of three exposed individuals at the end of the experiment. There were, therefore, no pathogen loads to quantify in the exposed tissue (Davy et al., 2017). We thank Field (2018) for confirming that *P. destructans* was virtually undetectable in the wing tissue of exposed *M. myotis,* as we also reported in our study.

## 4 ENVIRONMENTALLY PERSISTENT PATHOGENS PRESENT PARTICULAR RESEARCH CHALLENGES

In our view, some of Field's (2018) criticisms of our study are semantic in nature, which provides a welcome opportunity to explicitly consider terminology in studies of diseases such as WNS. Studies of wildlife diseases, including ours, often attempt to classify host–pathogen interactions into discrete categories. Hosts can be infected, naïve, or recovered; resistant, tolerant, or susceptible. These categories may work well for host‐transmitted diseases with pathogens carried internally in the host, because exposure and infection in these systems are more closely linked. However, environmentally persistent pathogens such as *P. destructans* present semantic challenges, because disease is a process that operates on a continuum.

Following exposure to a pathogen, a susceptible host may or may not become infected (i.e., develop clinical symptoms that can be confirmed by a pathologist). This distinction between exposure and infection is particularly relevant for environmentally persistent pathogens such as *P. destructans* or *Batrachochytrium dendrobatidis*, which can grow on some hosts without causing disease (Moore et al., 2017; Woodhams et al., 2007). If an exposed host does not develop detectable symptoms, it is impossible to determine, in hindsight, whether it was never infected or whether the symptoms were resolved before they became detectable. Where clinical infection occurs, experimental exposure studies typically point to the time of exposure as a meaningful baseline from which to measure the effects of infection. Is this appropriate in pathosystems such as WNS, where the pathogen may grow for some time on the host prior to causing disease? At what point in this process does a susceptible host that is exposed to an environmentally persistent pathogen qualify as “infected”? Discriminating among these definitions appears particularly challenging for environmentally persistent pathogens. However, this problem has also been identified in relation to microbial pathogens in clinical practice, where even advanced diagnostic methods cannot always identify the exact points at which exposure leads to infection (Pirofski & Casadevall, 2002).

Susceptibility, tolerance, or resistance to a pathogen also operate on a continuum. *Myotis lucifugus* are typically considered susceptible to *P. destructans* (Field et al., 2015; Warnecke et al., 2012), because mortality rates of naïve populations are high (>90%; Langwig et al., 2012, 2015). Yet populations of *M. lucifugus* persist in eastern North America, suggesting selection for resistance or tolerance (Donaldson et al., 2017; Langwig et al., 2017; Lilley et al., 2016). Should we consider free‐ranging *M. lucifugus* currently persisting in eastern North America as “susceptible”?

The line between tolerance and resistance to WNS is even blurrier. Pathogen loads and mild symptoms of WNS occur on free‐ranging *M. myotis* across its range, suggesting tolerance (Zukal et al., 2016). Yet in our study, we found no evidence of pathogen growth on this “tolerant” species (*M. myotis*), and no symptoms were observed, suggesting resistance. Is *M. myotis* a resistant species, a tolerant species, a “less‐susceptible” species, a species that is susceptible only under particular conditions, or a species with high interindividual variation in susceptibility?

Truly resistant species are still more elusive. The big brown bat (*Eptesicus fuscus*) is often labeled as resistant to WNS (Field, 2018; Frank et al., 2014; Moore et al., 2017). Yet 25% of “resistant” *E. fuscus* experimentally exposed to *P. destructans* developed clinical symptoms of WNS (Moore et al., 2017), and we strongly disagree with Field's (2018) classification of *E. fuscus* as a species that is resistant to WNS. Instead, the combined results of the studies mentioned here support a continuum of susceptibility, tolerance, and resistance to WNS in a range of species and that this continuum appears to be strongly context‐dependent.

Finally, our title was not intended to antagonize readers, but to highlight the diverse outcomes that can occur when bats of different species are exposed to *P. destructans*. The *M. lucifugus* in our experiment did develop infections that allowed us to characterize host and pathogen transcriptomes related to WNS, but we agree wholeheartedly that the responses of tolerant, resistant, or less‐susceptible species of bat to *P. destructans* merit further, detailed investigation.

The WNS system is proving to be extremely context‐dependent, and it will require the contributions of diverse research teams to disentangle it fully. We are grateful for this opportunity to further discuss the complexity of this system, which is shared by other emerging infectious diseases including snake fungal disease and chytridiomycosis in amphibians (Allender et al., 2015; Poorten & Rosenblum, 2016). Every study on these important wildlife pathogens has its strengths and limitations, but each study takes us another step closer to understanding and mitigating the impacts of these diseases on threatened wildlife populations. We encourage the research community to focus on understanding and finding solutions to the conservation challenges posed by emerging wildlife diseases.

## CONFLICT OF INTEREST

None declared.
